# Low oxygen content as a potential risk factor for scoliosis: a school-based cross-sectional study

**DOI:** 10.3389/fpubh.2026.1804995

**Published:** 2026-05-14

**Authors:** Jiahao Hao, Li Zhang, Zhi Zhao, Yingsong Wang, Shangjia Zhe, Fan Gao, Tao Li, Zhiyue Shi

**Affiliations:** Department of Orthopaedic, Second Affiliated Hospital of Kunming Medical University, Kunming, Yunnan, China

**Keywords:** altitude, hypoxia, oxygen content, scoliosis, screening

## Abstract

**Background:**

Scoliosis is a complex three-dimensional spinal deformity. While its clinical and imaging features are well established, the exact etiology and pathogenesis remain unclear. Previous studies have identified altitude as a significant environmental factor associated with scoliosis development. High-altitude regions are characterized by lower oxygen levels, raising the question of whether hypoxia contributes to the higher prevalence of scoliosis observed in these areas.

**Methods:**

This cross-sectional study used school-based scoliosis screening data. Individuals with an angle of trunk rotation (ATR) ≥ 5° were recorded as positive cases. Demographic information and regional environmental data were collected. Statistical analyses were conducted to identify risk factors associated with scoliosis.

**Results:**

The positive screening rate was 1.9%. Females and adolescents aged 10–18 years had significantly higher positivity rates than males and younger children (both *p* < 0.001). Han students showed slightly lower positivity compared with Bai and other minority groups (*p* = 0.001). Participants living above 2000 m and those in regions with oxygen content <235 g/m^3^ demonstrated higher positivity rates (both *p* < 0.001). Across all demographic subgroups, positivity increased with higher altitude and lower oxygen content. Further LASSO regression analysis showed that higher altitude and oxygen density below 235 g/m³ were positively associated with positive screening results, suggesting that individuals living in high-altitude, low-oxygen environments were more likely to screen positive(Coefficient=0.3527). In contrast, age 6–10 years was strongly negatively associated with positive screening results, indicating a relatively lower risk compared with other age groups(Coefficient = −1.3370). Correlation analysis further confirmed a significant negative association between environmental oxygen concentration and scoliosis detection rate (r = −0.784, *P* < 0.001).

**Conclusion:**

Lower ambient oxygen content is associated with higher scoliosis screening positivity.

## Introduction

Scoliosis is a complex three-dimensional spinal deformity that involves not only curvature in the coronal plane but also abnormalities in the sagittal and axial planes. It is clinically defined as a lateral spinal curvature exceeding 10° in the coronal plane, accompanied by axial vertebral rotation (1–3). During adolescence—a critical period of rapid growth and development—scoliosis can progress rapidly, potentially leading to cardiopulmonary dysfunction (4), chronic back pain, and even lower limb paralysis, thereby posing significant risks to both the physical and psychological well-being of affected individuals (5, 6). At present, surgical intervention remains the mainstay of treatment for severe scoliosis, with techniques such as posterior spinal fusion and the use of growth rods being commonly employed. However, these procedures are often associated with considerable trauma, extended recovery periods, and high financial costs. Therefore, early detection and timely, effective intervention are essential strategies to mitigate disease progression and significantly reduce the likelihood of requiring surgical treatment (7–9).

Previous studies have identified altitude as a significant environmental factor influencing the development of scoliosis (10–12). High-altitude environments are typically characterized by reduced ambient oxygen levels, which raises the question of whether hypoxic conditions contribute to the increased prevalence of scoliosis observed in these regions. This potential association between oxygen availability and scoliosis positivity represents a novel and emerging area of research.

As altitude increases, ambient oxygen levels generally decline. Prolonged exposure to hypoxic environments may elicit hypoxic stress responses in the human body, leading to the abnormal activation of reactive oxygen species (ROS) and hypoxia-inducible factors (HIF-*α*), which can disrupt normal physiological processes (13, 14). Under normal physiological conditions, bone resorption and formation remain in dynamic balance—a state known as bone homeostasis—that is essential for maintaining skeletal integrity. However, in hypoxic conditions, signaling pathways mediated by HIF-*α* and ROS have been shown to impair bone metabolism and compromise spinal stability (15–18).

At present, epidemiological data on scoliosis among primary and secondary school students in high-altitude regions of Southwest China remain limited. This study aims to conduct scoliosis screening among students across various regions of Yunnan Province, with the objective of identifying associated risk factors. The findings are expected to offer new insights that can inform strategies for early detection, timely intervention, and effective treatment of scoliosis in this population.

## Materials and methods

### Study population

This study conducted scoliosis screening among 181,801 primary and secondary school students in Dali Prefecture and Baoshan City. The cohort included 91,112 male and 90,689 female students. The average altitude of the screening areas was 1,977 meters above sea level, with an average atmospheric oxygen content of 234.1 g/m^3^.

### Screening methods

The screening team conducted on-site assessments at schools using a combination of visual inspection, the Adams Forward Bending Test (FBT), and measurement of the Angle of Trunk Rotation (ATR) with a scoliometer. Students with suspected positive findings were issued a scoliosis screening record form and a notification slip to document their basic information and preliminary screening results. Additionally, the altitude and corresponding atmospheric oxygen levels of the screening regions were measured to support environmental context analysis.

### Evaluation criteria

The evaluation involved a systematic assessment of physical symmetry, including observation of shoulder alignment, scapular symmetry, bilateral waistline symmetry, and equality in the height of the iliac crests. Additionally, the straightness of the line connecting the spinous processes was examined. Subsequently, the Adams Forward Bending Test was performed to detect any asymmetry in the posterior thoracic or lumbar regions. Finally, the Angle of Trunk Rotation (ATR) was measured using a scoliometer. An ATR measurement of ≥ 5° was considered a positive result in scoliosis screening.

### Data collection

Information was collected on each participant, including name, gender, age, ethnicity, and school, as well as the altitude and oxygen content of their location. The altitude of each screening site was measured using a portable altimeter (FR500, SUNROAD, China), and the recorded value was defined as the altitude of the screening location. The ambient oxygen concentration at each site was measured using an air oxygen analyzer (AR8100, Smart Sensor, China). Measurements were performed three consecutive times at each location, and the average value was calculated and recorded as the oxygen volume fraction of the ambient air at that screening site.

Subsequently, the oxygen density was calculated step by step according to the following formulas.

First, the ambient temperature at the corresponding altitude was estimated using the standard atmospheric lapse rate:


T=288.15−0.0065h

Where T represents temperature (K) and h represents altitude (m).

Second, the atmospheric pressure at the corresponding altitude was calculated using the barometric formula:


$$P=101325\times {\left(1\frac{0.0065h}{288.15}\right)}^{5.255}$$

Where P denotes atmospheric pressure (Pa).

Third, the partial pressure of oxygen was determined as:


$$P_{O2}=F_{O2}\times P$$

Where $F_{O2}$ represents the oxygen volume fraction in ambient air.

Finally, the oxygen density was calculated according to the ideal gas law:


$$\rho_{O2}=\frac{P_{O2}\times 0.032}{8.314\times T}\times 1000$$

Where $\rho_{O2}$ represents oxygen density (g/m^3^), 0.032 kg/mol is the molar mass of oxygen, and 8.314 J/(mol·K) is the universal gas constant.

### Statistical analysis

Statistical analyses were performed using SPSS version 26.0 (IBM Corp., Armonk, NY, USA) and Python. The detection rate was treated as a categorical variable and presented as frequencies and percentages. The study population was stratified by sex, age, and ethnicity. The chi-square (χ^2^) test was used to compare differences in detection rates among groups. The Cochran–Armitage trend test was applied to evaluate trends in detection rates across different altitude groups. In addition, Spearman correlation analysis was performed to assess the association between atmospheric oxygen concentration and the detection rate. Multicollinearity analysis was conducted to examine potential correlations among variables. LASSO regression analysis was then applied to identify factors associated with positive scoliosis screening results. A coefficient greater than 0 indicates that an increase in the corresponding factor is associated with a higher probability of a positive screening result, whereas a coefficient less than 0 indicates that an increase in the factor is associated with a lower probability of a positive screening result.

## Results

### Scoliosis positivity rates

- A total of 181,801 children and adolescents participated in this study, including 91,112 males and 90,689 females. Suspected scoliosis was detected in 3,616 individuals, resulting in an overall screening positivity rate of approximately 1.9%. The positivity rate in females (2.2%) was significantly higher than in males (1.7%, *p* < 0.001).
- The positivity rate in children over 10 years old (2.48%) was significantly higher than that in the 6–10 years age group (0.61%, *p* < 0.001).
- The positivity rate in Han ethnicity (1.8%) was slightly lower than in Bai ethnicity (2.08%) and other ethnic minorities (2.10%, *p* = 0.001).
- The positivity rate among individuals living at altitudes ≥ 2000 m (2.63%) was significantly higher than those living at altitudes < 2000 m (1.49%, *p* < 0.001).
- The positivity rate among individuals living in areas with an average oxygen content < 235.0 g/m^3^ (2.53%) was significantly higher than those in areas with an average oxygen content ≥ 235.0 g/m^3^ (1.31%, *p* < 0.001) (Table 1).

**Table 1:** Scoliosis positivity rates.

Item	Screening population	Positive cases	Positive rate	*P* value
Gender				
Male	91,112	1,579	1.73%	<0.001
Female	90,689	2037	2.25%	
Total	181,801	3,616	1.99%	
Age group				
6 ≤ Y < 10	48,069	293	0.61%	<0.001
10 ≤ Y < 19	133,732	3,323	2.48%	
Ethnicity				
Han	69,695	1,277	1.83%	0.001
Bai	60,758	1,262	2.08%	
Other Minorities	51,348	1,077	2.10%	
Residential altitude				
Altitude ≥ 2000	79,904	2098	2.63%	<0.001
Altitude < 2000	101,897	1,518	1.49%	
Average oxygen content				
Oxygen ≥ 235.0	80,442	1,052	1.31%	<0.001
Oxygen < 235.0	101,359	2,564	2.53%	

### Distribution of scoliosis positivity rates by oxygen content

#### Overall distribution

**Figure 1:**
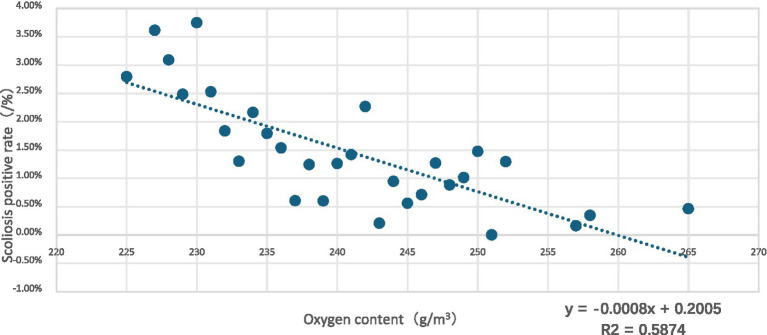
Distribution of scoliosis positive rate among children and adolescents with respect to oxygen content.

Analysis showed that scoliosis positivity rates decreased with increasing oxygen content (R^2^ = 0.5874)(Figure 1).

### Distribution in subgroups by gender, age, and ethnicity

**Figure 2:**
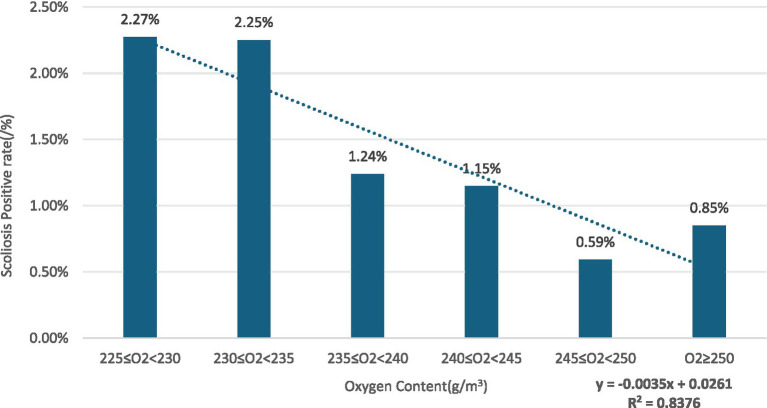
Scoliosis rate in young males versus oxygen content.

**Figure 3:**
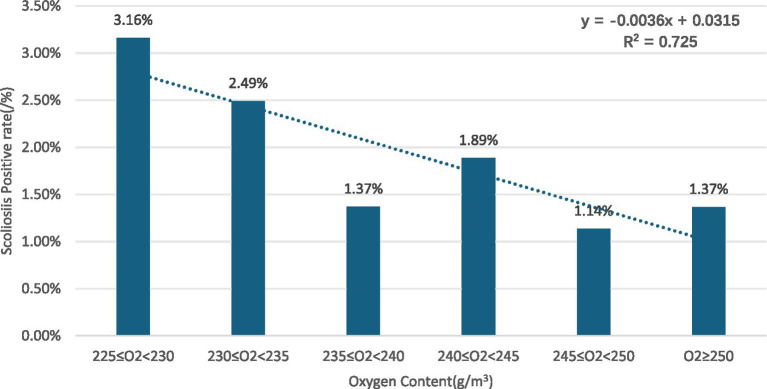
Scoliosis rate in young females versus oxygen levels.

**Figure 4:**
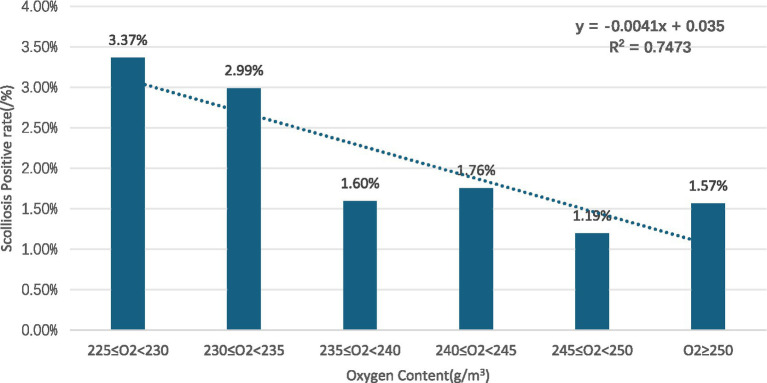
Scoliosis rate in adolescents (10+) versus oxygen content.

**Figure 5:**
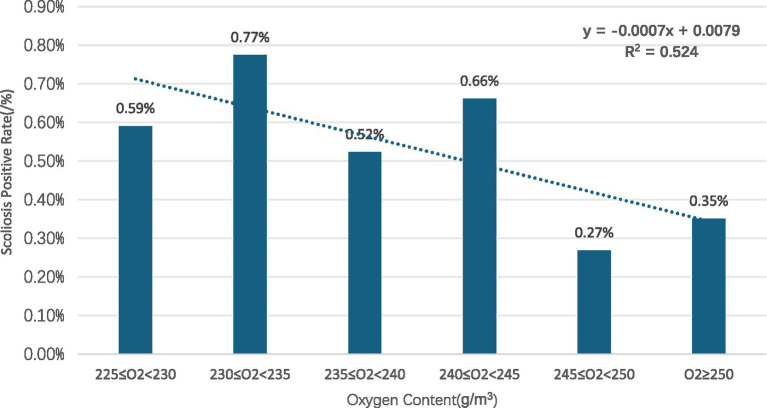
Scoliosis positive rate in children under 10 versus oxygen content.

**Figure 6:**
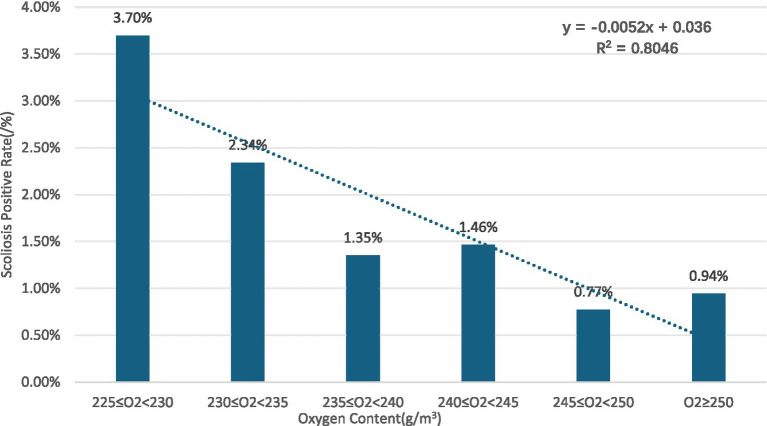
Relationship of scoliosis positivity with oxygen content in Han children and adolescents.

**Figure 7:**
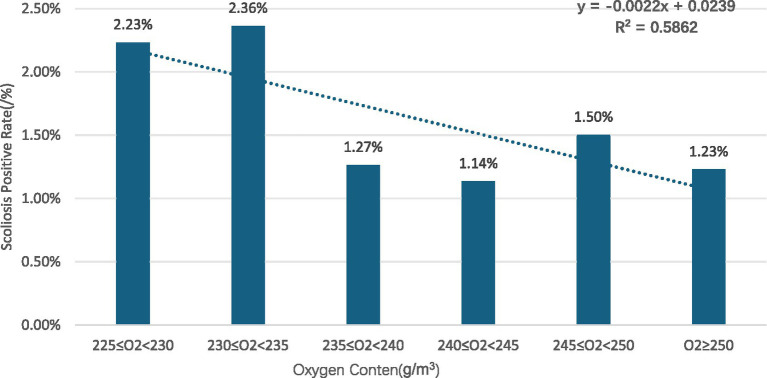
Relationship of scoliosis positivity with oxygen content in Bai children and adolescents.

**Figure 8:**
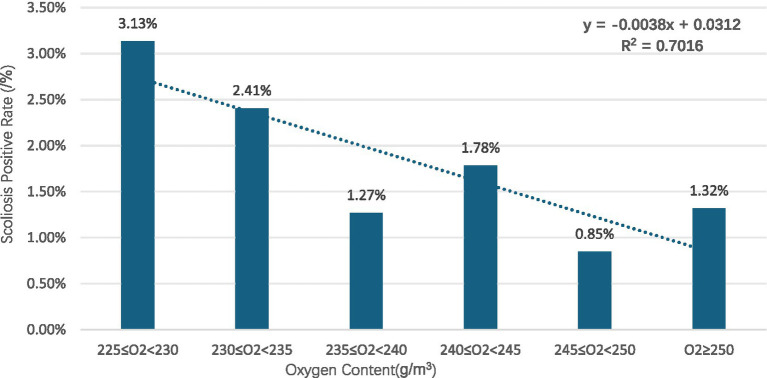
Scoliosis prevalence in other ethnicity youth versus oxygen content.

- Gender subgroups: in both males and females, scoliosis positivity rates showed an overall decreasing trend with increasing oxygen content for males (R^2^ = 0.8376) (Figure 2) and for females (R^2^ = 0.725) (Figure 3).
- Age subgroups: positivity rates decreased with increasing oxygen content for both individuals over 10 years old (R^2^ = 0.7473) (Figure 4) and those under 10 years old (R^2^ = 0.524) (Figure 5).
- Ethnicity subgroups: among Han (R^2^ = 0.8046) (Figure 6), Bai (R^2^ = 0.5862) (Figure 7), and other ethnic groups (R^2^ = 0.7016) (Figure 8), scoliosis positivity rates also generally decreased as oxygen content increased.

### Distribution of scoliosis positivity rates by altitude

#### Overall distribution

**Figure 9:**
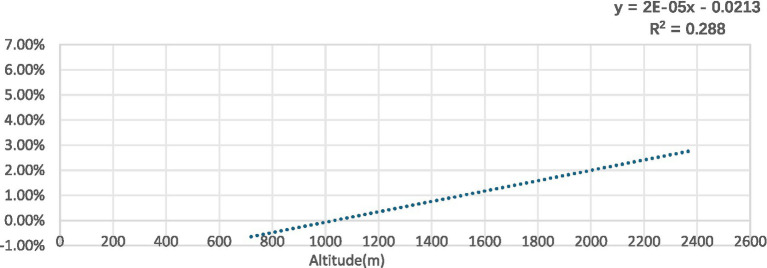
Scoliosis prevalence in children and adolescents by altitude.

Analysis revealed that scoliosis positivity rates increased with increasing altitude (R^2^ = 0.288) (Figure 9).

#### Subgroups by gender, age, and ethnicity

**Figure 10:**
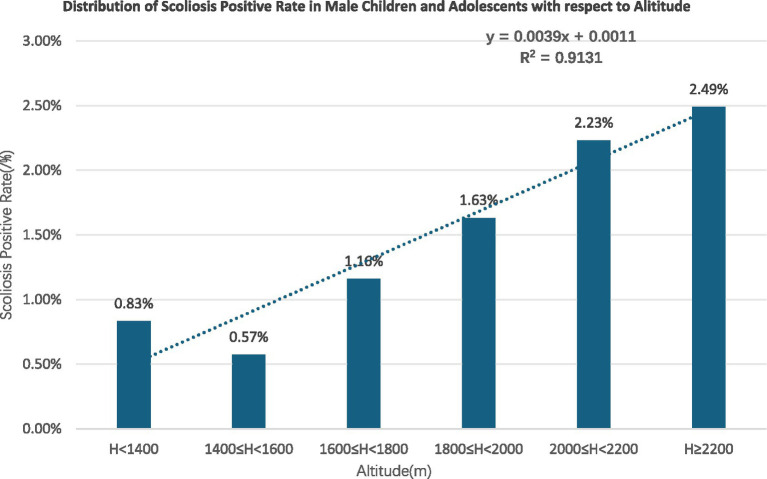
Scoliosis rate distribution in boys versus altitude.

**Figure 11:**
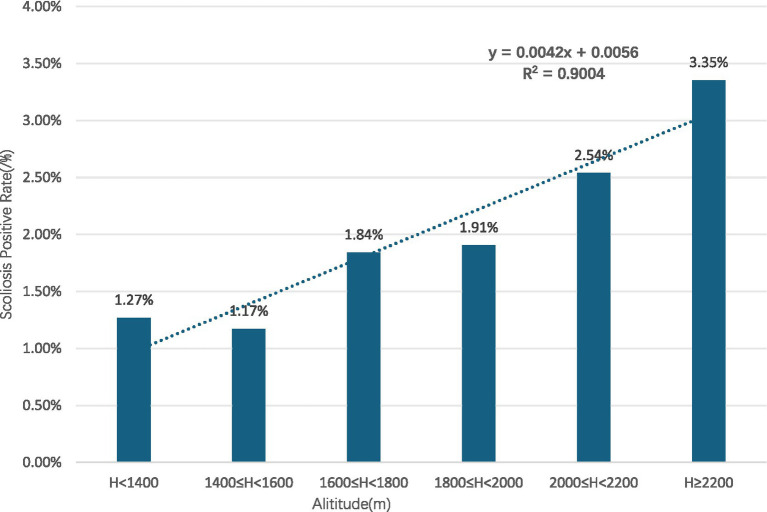
Scoliosis positive rate in female youth by altitude.

**Figure 12:**
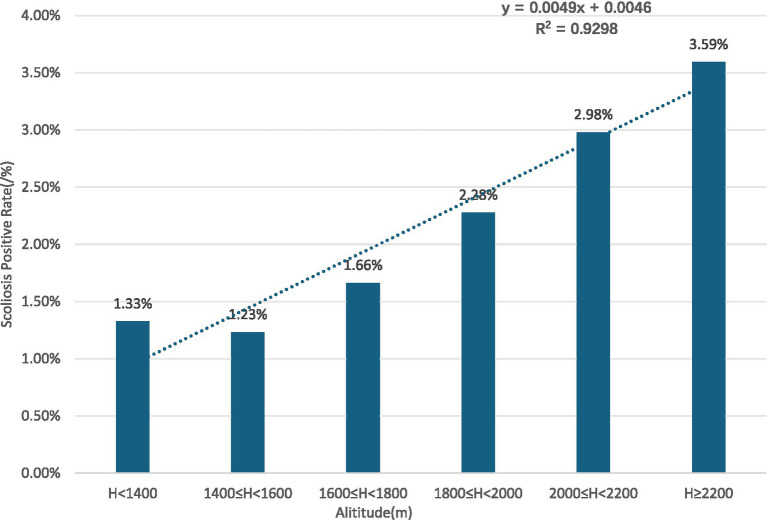
Scoliosis positive rate distribution in youth over 10 by altitude.

**Figure 13:**
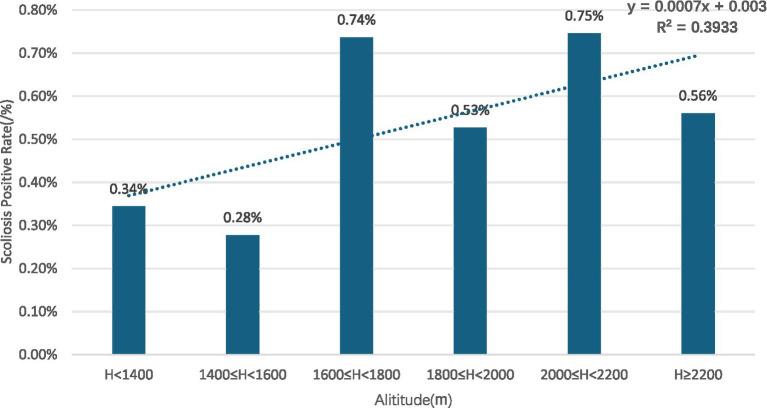
Scoliosis positive rate distribution in children under 10 by altitude.

**Figure 14:**
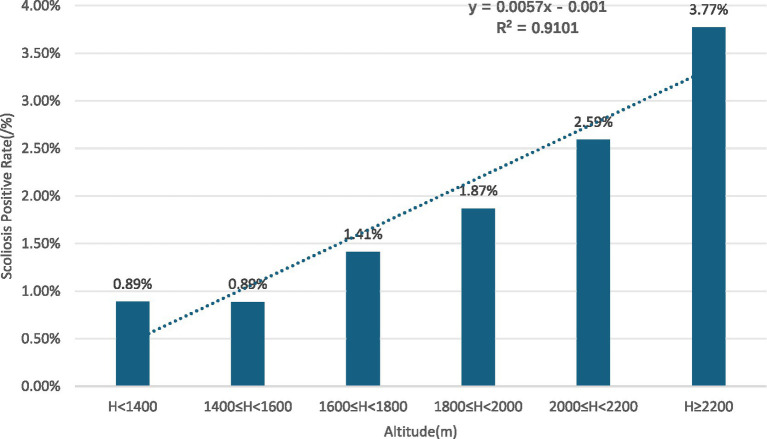
Scoliosis prevalence in Han children and adolescents by altitude.

**Figure 15:**
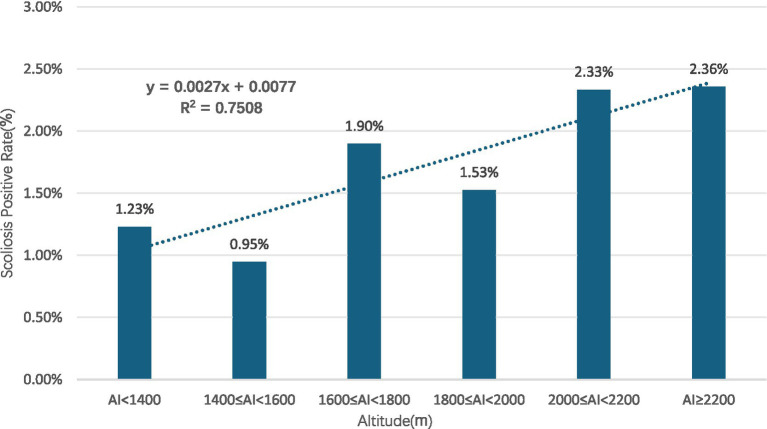
Scoliosis prevalence in Bai children and adolescents across altitudes.

**Figure 16:**
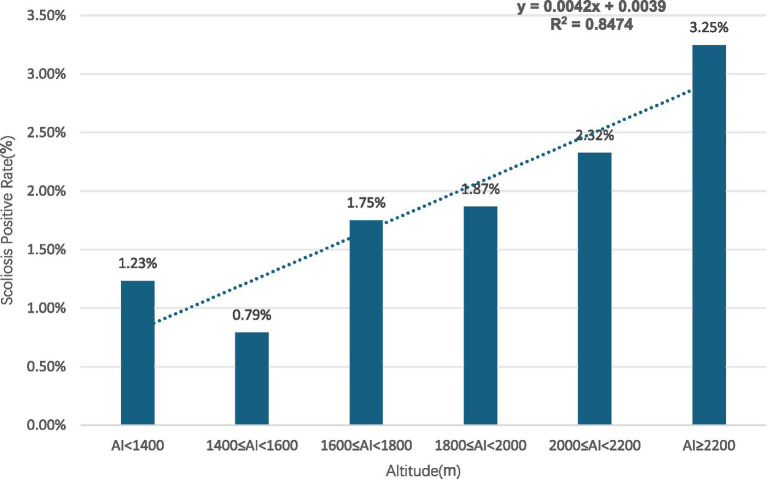
Scoliosis rate in other ethnic children and adolescents by altitude.

- Gender subgroups: both male and female children and adolescents showed an increasing trend in scoliosis positivity rates with rising altitude (R^2^ = 0.9131 for males, R^2^ = 0.9004 for females) (Figures 10, 11).
- Age subgroups: scoliosis positivity rates increased with rising altitude in both children over 10 years old (R^2^ = 0.9298) (Figure 12) and those under 10 years old (R^2^ = 0.3933) (Figure 13).
- Ethnicity subgroups: scoliosis positivity rates among Han (R^2^ = 0.9101) (Figure 14), Bai (R^2^ = 0.7508) (Figure 15), and other ethnic minorities (R^2^ = 0.8474) (Figure 16) also showed an overall increasing trend with altitude.

### Factors influencing scoliosis positivity

**Figure 17:**
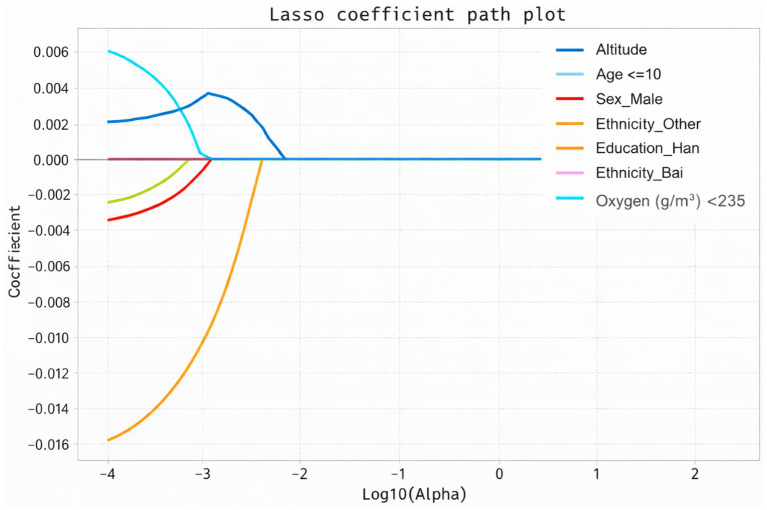
Coefficient profiles of the LASSO model.

**Figure 18:**
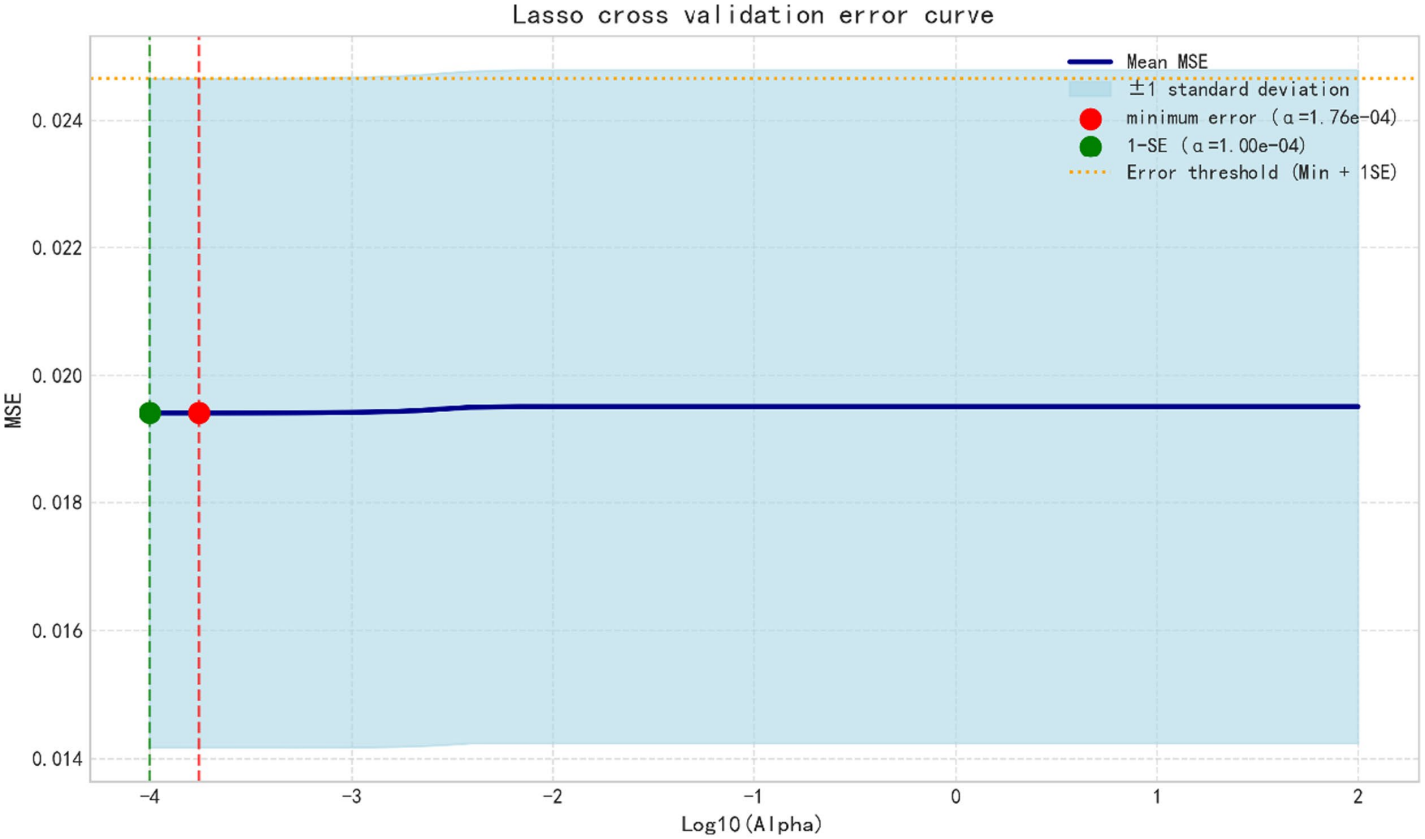
Cross-validation curve for the LASSO model.

**Figure 19:**
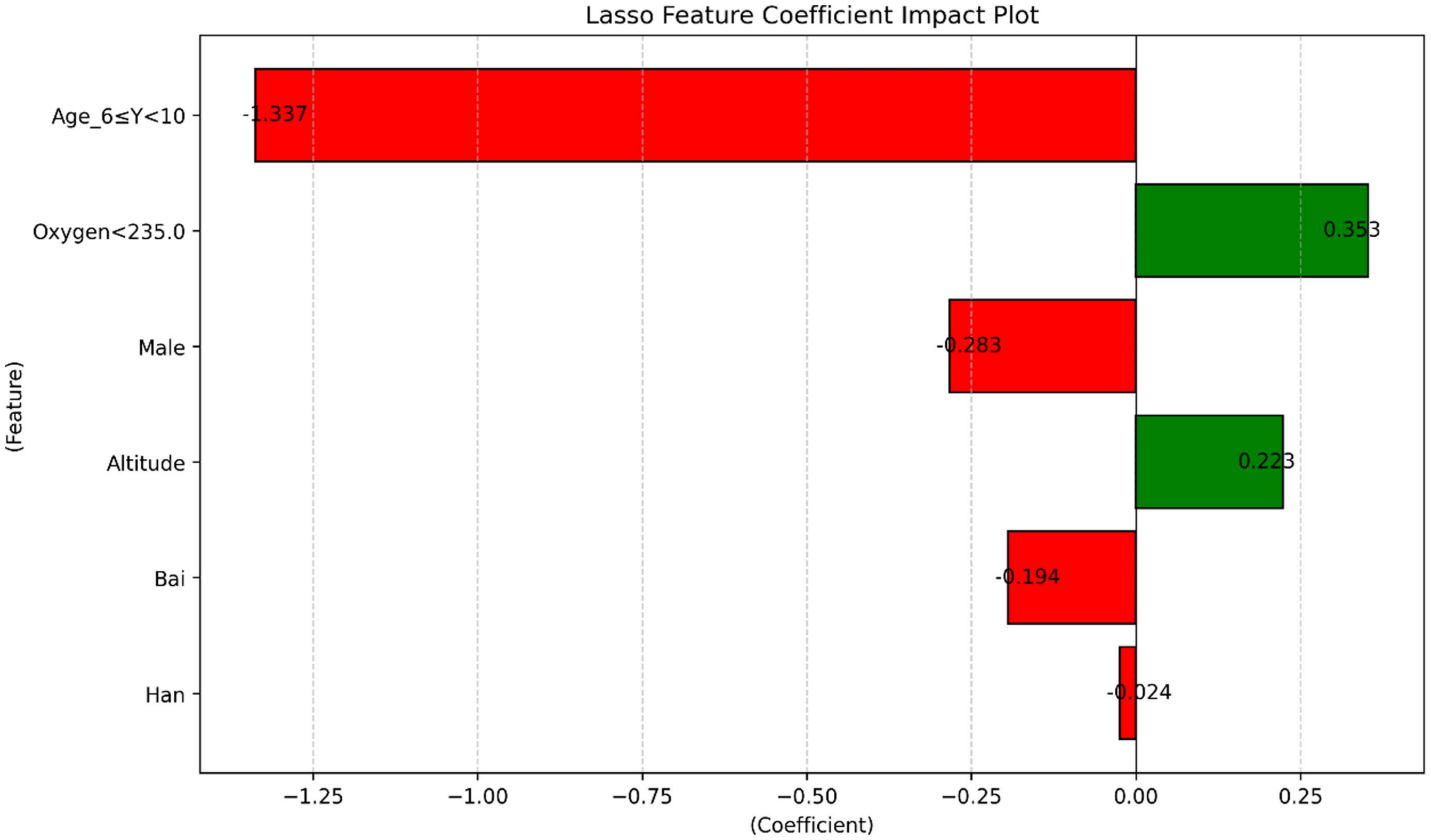
Feature importance plot based on the LASSO regression mode.

Altitude was significantly positively associated with the screening outcome (*β* = 0.365, t = 7.072, *p* < 0.001). Similarly, oxygen density was also significantly positively associated with the screening outcome (*β* = 0.321, t = 6.229, *p* < 0.001). Collinearity diagnostics indicated extremely high variance inflation factors (VIF = 484.583) and very low tolerance values (0.002), suggesting the presence of severe multicollinearity between the predictors (Table 2). To identify factors associated with positive scoliosis screening results, LASSO logistic regression analysis was performed. After applying L1 regularization, variables with coefficients equal to zero were excluded, and six variables with non-zero coefficients were retained in the final model. The results indicated that higher altitude and oxygen density below 235 g/m^3^ were positively associated with positive screening outcomes, suggesting that individuals living in high-altitude environments with lower oxygen density were more likely to screen positive. Age was identified as the most influential factor. Specifically, children aged 6–10 years showed a strong negative association with positive screening results, indicating a lower risk compared with other age groups. In addition, demographic characteristics, including sex and ethnicity, were also associated with screening outcomes. Male sex and Bai ethnicity were found to have a protective effect in the model. Overall, these findings suggest that both environmental factors (altitude and oxygen) and demographic characteristics influence the likelihood of a positive scoliosis screening result (Table 3) (Figures 17–19), were identified as risk factors for scoliosis. Correlation analysis showed a significant negative correlation between environmental oxygen content and scoliosis detection rate (r = −0.784, *p* < 0.001) (Table 4).

**Table 2:** Collinearity diagnostics.

Variable	B	Std.	β	t	*p*	Tolerance	VIF
Altitude	0.000	0.000	0.365	7.072	<0.001	0.002	484.583
Oxygen (g/m^3^)	0.007	0.001	0.321	6.229	<0.001	0.002	484.583

**Table 3:** LASSO regression analysis.

Feature	Coefficient	Absolute_Coefficient
Age_6 ≤ Y < 10	−1.337007	1.337007
Oxygen(g/m^3^)<235	0.352714	0.352714
Male	−0.283419	0.283419
Altitude	0.222675	0.222675
Bai	−0.194350	0.194350
Han	−0.023818	0.023818
Other Nations	0.000000	0.000000

**Table 4:** Analysis of correlations in scoliosis positivity rate.

Variable	Altitude	Oxygen content
Spearman’s ρ	0.556	−0.784
*P* value	*P* < 0.001	*P* < 0.001

## Discussion

From an etiological perspective, scoliosis can be broadly classified into congenital scoliosis, idiopathic scoliosis, syndromic scoliosis, and degenerative scoliosis. Among these subtypes, idiopathic scoliosis is the most prevalent. Because the condition most commonly develops during adolescence, it is generally referred to as adolescent idiopathic scoliosis (AIS). Despite extensive research, the exact pathogenesis of AIS remains incompletely understood. Previous studies have proposed multiple potential mechanisms, including genetic susceptibility, musculoskeletal and biomechanical abnormalities, endocrine dysregulation, neural control factors, disturbances in growth and development, and environmental influences.

In the present study, the average altitude of the study region was 1,977 m, with a mean oxygen density of 234.1 g/m^3^. The screening-positive rate of scoliosis among children and adolescents in Dali Prefecture and Baoshan City of Yunnan Province was approximately 1.9%. This prevalence is comparable to the rate reported in Shanghai (2.00%) (11) but lower than those reported in Gansu Province (3.69%) (12) and the Qinghai–Tibet Plateau (5.68%) (19). However, differences in screening-positive rates across regions may reflect multiple underlying factors, including variations in geographic environments, demographic characteristics, screening procedures, threshold criteria, and unmeasured confounding variables. Therefore, these regional differences should not be attributed to a single environmental factor.

Within our study population, we observed a trend in which the screening-positive rate of scoliosis increased with rising altitude. As altitude increases, oxygen availability generally decreases. Previous studies by Jiang et al. (20) and Hou et al. (21) have reported that children and adolescents living in high-altitude, hypoxic environments may exhibit certain physiological indicators that are lower than those observed in individuals residing at lower altitudes. These studies also documented altitude-related differences in scoliosis detection rates. Collectively, these findings suggest that exposure to lower oxygen levels in high-altitude regions may be statistically associated with a higher screening-positive rate of scoliosis. Nevertheless, this association should be interpreted cautiously and requires further confirmation through studies incorporating more comprehensive confounder control and longitudinal designs.

Our subgroup analyses further demonstrated that, after stratification by sex, age, and ethnicity, the screening-positive rate of scoliosis tended to increase as oxygen density decreased. Scoliosis fundamentally represents a disorder of spinal growth and structural stability. Abnormalities in osteogenesis and bone metabolic regulation have been proposed as potential contributors to the development and progression of AIS. Bone marrow mesenchymal stem cells (BMSCs) play a critical role in skeletal development and bone metabolism because of their multipotent differentiation capacity. These cells can differentiate into various skeletal lineage cells, thereby contributing to bone formation and maintaining skeletal homeostasis (22, 23). Recent studies have suggested that functional abnormalities in BMSCs may be closely associated with the pathogenesis and progression of AIS. Ko DS summarized the roles of genes, proteins, and non-coding RNAs expressed in BMSCs in AIS pathogenesis, suggesting that dysregulated BMSC function may contribute to disease development (24). Furthermore, individuals with AIS have frequently been reported to exhibit systemic low bone mass, reduced bone mineral density in both axial and peripheral skeletons, and disproportionate endochondral and membranous bone growth. Lower bone density has also been identified as a potential risk factor for curve progression in AIS (25).

Oxygen is an essential environmental factor required for maintaining normal cellular metabolism and physiological processes. Alterations in oxygen availability may influence cellular metabolic pathways and functional states. Reduced oxygen levels are generally associated with increased oxidative stress, accelerated cellular senescence, and inhibition of cell proliferation and differentiation (26, 27). Hypoxic conditions may also affect the biological functions of BMSCs and regulate their proliferation and differentiation through signaling pathways such as Runx2, Sox9, Wnt, and PI3K/Akt (28, 29). Disruption of these regulatory pathways may ultimately alter bone metabolic balance and skeletal structural stability. Taken together, these findings suggest a potential pathogenic pathway under hypoxic conditions, involving hypoxia-induced dysfunction of BMSCs and subsequent bone metabolic imbalance, which may contribute to the onset and progression of AIS.

In addition to bone metabolism, paraspinal muscles also play a critical role in maintaining spinal stability (30, 31). Some studies have suggested that hypoxia-related physiological changes may influence the expression of hypoxia-inducible factor (HIF-*α*) and the levels of reactive oxygen species (ROS), thereby affecting paraspinal muscle function. Such alterations may lead to asymmetric mechanical loading between the two sides of the spine, resulting in reduced spinal stability and potentially contributing to the development of scoliosis (32, 33). Furthermore, a clinical study conducted by Ugur reported that scoliosis improved after adenoidectomy in patients with adenoid hypertrophy accompanied by scoliosis, suggesting a potential link between hypoxia-related conditions and scoliosis progression (34). In the specific geographic context of the Yunnan–Guizhou Plateau, the observed association between reduced oxygen availability and increased scoliosis screening-positive rates may therefore involve multiple biological pathways, including bone metabolism, skeletal growth, and paraspinal muscle function. Nevertheless, these mechanisms remain hypothetical and require validation through experimental research and prospective cohort studies.

Our findings also indicated that the screening-positive rate of scoliosis was significantly higher in females than in males, which is consistent with previous reports (35). Females typically enter puberty earlier and experience more rapid growth spurts compared with males. During puberty, increased estrogen levels may bind to estrogen receptors (ESR1 and ESR2) and potentially induce receptor methylation, which may contribute to asymmetric development of paraspinal muscles and alterations in spinal stability (36–38). Age-related differences were also observed, with children and adolescents aged over 10 years demonstrating a significantly higher screening-positive rate compared with younger individuals. Previous studies examining the risk of AIS progression have suggested that the adolescent growth spurt represents a critical period for the onset and progression of scoliosis (39). Rapid physical growth during puberty, accompanied by substantial hormonal fluctuations, may disrupt the coordination of endochondral ossification and vertebral growth, thereby increasing the risk of spinal imbalance and scoliosis development (40). These findings further highlight the importance of early screening and timely intervention during the pubertal growth phase to reduce the risk of rapid curve progression and improve long-term outcomes.

Several limitations of this study should also be acknowledged. The association observed between regional oxygen availability (or altitude) and scoliosis screening-positive rates may be influenced by multiple regional factors. Socioeconomic status, nutritional intake, growth and developmental conditions, patterns of physical activity, sedentary behavior, schoolbag weight, and the ergonomic suitability of school furniture may all affect spinal biomechanical loading in children and adolescents, thereby contributing to the development of scoliosis (41–43). In high-altitude regions in particular, relatively inadequate nutritional intake or restricted growth may partially explain higher screening-positive rates, independent of hypoxic exposure. In the present study, these potential confounding factors were not comprehensively measured or fully adjusted for. Future studies should incorporate more detailed individual-level data collection, improved exposure assessment, and longitudinal study designs to better clarify the independent association between oxygen availability and scoliosis risk and to further explore the underlying biological mechanisms.

## Conclusion

Reduced atmospheric oxygen availability may represent a potential environmental risk factor associated with higher screening-positive rates of scoliosis. Hypoxic exposure may alter the physiological functions of bone marrow mesenchymal stem cells (BMSCs), potentially leading to dysregulated bone formation, imbalanced skeletal development, and dysfunction of paraspinal muscles. These processes may form a potential pathogenic cascade involving hypoxia, cellular dysfunction, and disruption of bone metabolic homeostasis, thereby contributing to the development and progression of scoliosis. Moreover, altitude, sex, and age were also identified as important factors associated with the screening-positive rate of scoliosis. Collectively, these findings may provide new insights into the early detection, prevention, and management of scoliosis.

### Limitations and future directions

This study has several limitations. First, the screening was conducted in school settings without radiographic confirmation, which may limit the accuracy of the estimated prevalence of scoliosis. Second, regional differences in population structure, socioeconomic status, and lifestyle patterns may have introduced potential confounding and statistical bias. These factors were not comprehensively measured or fully adjusted for in the present study. Future studies should incorporate more detailed individual-level data collection, improved exposure assessment, and longitudinal study designs. Finally, because this study employed a cross-sectional design, it was only able to identify potential risk factors associated with scoliosis rather than establish causal relationships. Further longitudinal and mechanistic studies are therefore required to clarify the underlying pathophysiological mechanisms.

## Data Availability

The raw data supporting the conclusions of this article will be made available by the authors, without undue reservation.
